# Towards a neuroethological approach to consciousness

**DOI:** 10.1098/rstb.2024.0307

**Published:** 2025-11-13

**Authors:** Yuranny Cabral-Calderin, Julio Hechavarria, Lucia Melloni

**Affiliations:** ^1^ Research Group Neural Circuits, Consciousness and Cognition, Max Planck Institute for Empirical Aesthetics, Frankfurt am Main, Hessen 60322, Germany; ^2^ AG Brain and Behavior, Department of Biology, Freie Universität Berlin, Berlin, Berlin 14195, Germany; ^3^ Hechavarría Lab, Ernst Strüngmann Institute for Neuroscience in Cooperation with Max Planck Society, Frankfurt am Main, Hessen 60528, Germany; ^4^ Department of Neurology, Grossman School of Medicine, New York, NY 10017, USA; ^5^ Predictive Brain Department, Research Center One Health Ruhr, University Alliance Ruhr, Ruhr-Universität Bochum, Bochum 44801, Germany

**Keywords:** consciousness, infants, animal cognition

## Abstract

Understanding consciousness remains a significant challenge in science. What distinguishes conscious beings from unconscious systems, such as organoids, artificial intelligence or other non-sentient entities? Research on consciousness often focuses on identifying brain activity associated with conscious and non-conscious states, primarily in neurotypical human adults. However, this approach is limited in scope when applied to entities with developmental or evolutionary trajectories different from our own. How do we investigate consciousness in infants, whose brains are still maturing or in non-human animals, shaped by diverse ecological and evolutionary pressures? This opinion piece encourages consciousness studies to adopt a neuroethological perspective, drawing on Tinbergen’s framework for studying behaviour. By examining the (1) mechanisms, (2) development, (3) adaptive functions and (4) evolutionary origins of consciousness, we can move beyond a human-centric focus to explore its diversity across life forms. Most investigators now accept that consciousness is not confined to humans alone but that some other animals have it, and it is a continuum shaped by evolutionary pressures. By adopting this broader approach, consciousness studies can better investigate and understand consciousness in its various forms and contexts, with significant scientific, ethical and societal implications.

This article is part of the theme issue ‘Evolutionary functions of consciousness’.

## 1. Introduction

A peculiar narrowness of modern human thinking is indicated by the fact that relatively few biologists seem to be willing to give their attention to both the causes and the effects of observed life processes. Many of them confine themselves to the study of the causes underlying the observed phenomena, as do the majority of physiologists, others prefer to study the way in which life processes contribute to the maintenance of life, as do most ecologists.—Nikolaas Tinbergen [1, p. 188]

Consciousness remains one of science’s greatest enigmas. While remarkable progress has been made in understanding the origins of the universe, the nature of life and the mechanisms of evolution, the question of how the brain generates subjective experiences—feelings, thoughts and awareness—remains unresolved. *Why does consciousness arise and what purpose does it serve?*

To date, much of the research has largely focused on identifying the neural correlates of consciousness, typically in neurotypical human adults [2]. These studies often rely on psychophysical paradigms that manipulate sensory stimuli—making them perceptually ambiguous or invisible—and contrast brain activity patterns associated with conscious versus unconscious perception. While this contrastive methodology has yielded valuable insights, it faces critical limitations that constrain its scope [3,4] and generalizability [5].

One major limitation lies in its dependance on subjective reports, mainly verbal, to validate conscious experiences. This approach becomes problematic when studying populations that cannot communicate overtly or whose cognitive functions diverge from the adult norms (e.g. short or long memory span or capacity for abstraction), such as infants, non-human animals or patients with severe neurological impairments. Behavioural proxies for consciousness grounded in adult, neurotypical cognitive systems can easily under- or overestimate consciousness in different developmental stages and also across organisms. This approach raises profound ethical and methodological questions: how do we ensure the accuracy of these assessments when inferring consciousness in populations in which report is limited?

Moreover, the neuroscientific study of consciousness has often overlooked the natural contexts in which behaviours—and potentially consciousness—arise. Conscious processes do not occur in isolation; they emerge from the organism’s interaction with its environment. A neuroethological approach, which examines the evolutionary and functional purposes of behaviour, offers a more comprehensive understanding. This perspective considers the stimuli and conditions that shape consciousness and its role in adaptive behaviours, including in the complex social interactions of animals. Thus, this approach allows us to ask not only *whether* an organism is conscious but also *why* it might be conscious in a particular context, and *how* that consciousness supports survival and behaviour. By adopting this approach, we can move beyond an adult, neurotypical, human-centred view of consciousness to explore its variations across ontogeny and phylogeny.

Neglecting these variations would be akin to trying to understand Earth while ignoring its 4.5 billion years of history and cosmic context. Consciousness, too, must be understood as a dynamic and evolving phenomenon. Investigating its gradations across developmental stages and species is crucial for ethical, scientific and practical reasons. For instance, better tools to measure consciousness could inform critical care decisions, animal welfare policies and even the ethical development of artificial intelligence.

To address this, we propose a framework grounded in Tinbergen’s four questions—mechanism, function, ontogeny and phylogeny—as a guiding structure for consciousness research, which offers a new perspective to advance our understanding of consciousness, i.e. how and why consciousness is instantiated in physical and biological systems [6]. These principles, long central to neuroethology (i.e. the study of the neural mechanisms underlying behaviour [7]), have greatly advanced the study of behaviours such as navigation, fight/flight responses, fear and aggression, by uncovering context-specific neural mechanisms and adaptive functions. A similar approach could illuminate the functions and neural underpinnings of consciousness, shifting the focus from what consciousness is to what consciousness does, and how that contribution may vary across ontogeny and phylogeny. Crucially, the Tinbergen framework imposes coherence across explanatory levels: mechanistic accounts must align with functional roles and be consistent with ontogenetic and phylogenetic constraints. In doing so, it promotes a more inclusive scientific posture that acknowledges the possible variants of consciousness, treating human consciousness as one possible instantiation among many, rather than the gold standard.

This article outlines a roadmap for a neuroethological approach to consciousness. We begin by defining consciousness, then outline Tinbergen’s framework to assess the presence of consciousness beyond adult humans. Following this framework, we discuss whether mechanisms from human theories extend to other species, explore the possible functions of consciousness and review evidence from newborns, fetuses and non-human animals. Finally, we demonstrate how Tinbergen’s framework can help to uncover the nature of consciousness across developmental stages and species. The purpose of this review is to lay the groundwork for a more integrative and biologically grounded science of consciousness, one that moves beyond the confines of neurotypical adult humans to embrace the full richness and diversity of conscious experience within humans (by considering development) and across the animal kingdom.

## 2. What do we mean by consciousness?

A unified and universally accepted definition of consciousness remains elusive. For the purpose of our neuroethological approach, we consider useful the distinction articulated by Block [8] between *phenomenal consciousness* (*P-consciousness*) and *access consciousness* (*A-consciousness*). *P-consciousness* refers to the subjective, experiential qualities of consciousness—the raw ‘what it is like’ to experience something [9]. This dimension of consciousness highlights sentience: the capacity to have subjective experiences, such as pain, pleasure or sensory perception. In contrast, *A-consciousness* refers to the cognitive processes mediated by consciousness, e.g. making information accessible for reasoning, decision making and guiding behaviour [8,10,11]. *A-consciousness* emphasizes functionality—how mental states are accessed, reported and acted upon—making it central to discussions of cognition and metacognition. In this framework, we consider both *P-consciousness* and *A-consciousness*. We hypothesize that *A-consciousness* builds on *P-consciousness*, allowing us to infer the presence of *P-consciousness* through observations of *A-consciousness* across development and species, while also allowing for a dissociation between the two and the possibility of *P-consciousness* being present on its own. This approach enables us to explore the potentially distinct functional advantages of each, with *P-consciousness* providing a preserved experiential basis and *A-consciousness* reflecting species-specific cognitive elaborations.

## 3. Extending the study of consciousness beyond the neurotypical human adult

In this work, we adopt an evolutionary, neuroethological perspective on consciousness, proposing that it emerged gradually through natural selection, rather than arising de novo in humans [12]. We adhere to the continuity hypothesis—first articulated by Darwin and supported by subsequent thinkers such as George Romanes and Even Lloyd Morgan—which assumes that both *P-consciousness* and *A-consciousness* exist on a spectrum across species, with complex forms building upon more basic experiential capacities [13]. Nervous systems and behaviours differ widely across species, shaped by distinct ecological pressures and cognitive demands. This diversity raises a foundational question: which species are conscious, and how can we tell?

We argue that searching for human-like signatures of consciousness—such as verbal report—is a flawed approach. This strategy risks underestimating consciousness in species that cannot share their mental states through verbal report, while simultaneously overestimating it in artificial systems (e.g. large language models) that can produce verbal outputs but lack biological details, ecological grounding and a history of co-evolution. Instead, following Andrews [14–16], we advocate for a shift in baseline assumptions. Rather than starting with the default that only human-like minds are consciousness, we shift the assumption to one where many animals—including those only distantly related to humans—possess forms of consciousness adapted to their specific biological and environmental contexts. This perspective aligns with a historically continuous view of consciousness—dating back to Socrates and embraced by Darwin—which held that human and non-human minds differ in degree rather than kind. To move the field forward, we propose four guiding questions—adapted from Tinbergen’s classic framework—to structure the investigation of consciousness across stages of development and species: (1) What neural mechanisms underlie consciousness in a given animal (causation/mechanisms)? (2) What adaptive functions does consciousness serve within specific ecological niches (function/adaptation)? (3) How does consciousness emerge and change over an individual’s lifetime (ontogeny/development)? and (4) Is consciousness present in other closely or distantly related species (phylogeny/evolution)?

These questions, known as Tinbergen’s four questions (Box 1, [6]) were originally postulated to guide the study of animal behaviour, but they can be effectively applied to any biological trait, including consciousness. They provide a structured framework for explanation along two complementary axes. Questions 1 and 3 (causation and ontogeny) provide *proximate* answers that evaluate *how* consciousness operates in single individuals, from newborns to adults. Questions 2 and 4 (function and phylogeny) could provide an *ultimate* (evolutionary) view of consciousness that considers *why* consciousness is evolutionarily beneficial. Studies around the mechanisms and functions of consciousness are *static* in the sense that they provide a view of consciousness in its current form for a given species. On the other hand, studies focusing on ontogeny and phylogeny are *dynamic* in the sense that they reveal how consciousness changes across multiple timescales. We propose using Tinbergen’s four questions as a formal framework to help overcome the current human-centred approach to consciousness research, thereby broadening the scope of the discipline and guiding both research and theory development. At present, the field has focused almost exclusively on Question 1—mechanisms—which has also been the emphasis of most theories of consciousness, often at the expense of Questions 2–4 [17]. This narrow focus has consequently limited the scope and applicability of these theories. The proposed framework seeks ultimately to help us identify what constitutes an appropriate and ecologically valid test for evaluating consciousness in models other than in neurotypical human adults. In what follows, we expand on how the field of consciousness can benefit from an approach inspired by Tinbergen’s explanatory levels.

Box 1. **Tinbergen’s four questions adapted to consciousness research**

**Table:** 

	static view current form for a given species	dynamic view current form in terms of a historical sequence
**proximate** (how?)	**causation/mechanisms** what neural mechanisms allow a given animal to be conscious?	**ontogeny/development** how does consciousness develop in single individuals?
**ultimate** (why?)	**function/adaptation** what functions does consciousness serve within specific ecological niches?	**phylogeny/evolution** is consciousness present in other evolutionarily close species?

## 4. Causal mechanisms of consciousness

The first question of the Tinbergen-based approach asks what neural mechanisms cause a given animal or developmental stage to be conscious. To address this, as a starting point, we propose leveraging existing theories of human consciousness (for comprehensive reviews see [18–21]) to test whether their same markers exist in animals. These include, for example, markers like the recurrent cortical processing proposed by the Recurrent Processing Theory (RPT [22]), the ignition dynamics central to Global Neuronal Workspace Theory (GNWT [23–25]), the temporally coordinated feedback loops described by Predictive Processing Theory (PPT [26,27]) or pleasure hot spots and cold spots within the subcortical brain regions that humans share with all other vertebrates—spots whose existence is predicted by the Emergent System Theories (ESTs [28–31]; also see [32]), to name a few. Additionally, as the mechanisms of adult human consciousness are tested and validated, researchers can use the same investigative techniques in fetuses and animals: e.g. electroencephalography, functional MRI and electrical recording from the neuronal networks that are suspected to be involved in consciousness.

Importantly, most evidence about the neural mechanisms of consciousness in humans suffers from important shortcomings: for instance, evidence is still correlative in nature, contaminated by various confounds, such as motor reports, and has focused mostly on the visual modality narrowing the understanding of the brain basis of consciousness in other sensory modalities. For instance, the involvement of frontal regions in conscious access, as predicted by different theories like GNWT and variants of Higher-Order Thought Theories [24,25,33], has been questioned by studies showing diminished frontal activity when no motor report is required [34]. Therefore, establishing the neural mechanisms of consciousness in humans and beyond will require the development of carefully controlled methodologies that can account for and remove confounding variables as well as perturbation-based approaches to prove their causal involvement [35,36]. In the past, we have advocated for a strategy to distil theneural correlates of consciousness that focuses on neural signatures that are stable across stimulus manipulations, report and no-report paradigms and experimental tasks [3]. This approach remains especially promising today, given the growing trend towards data sharing in the field. The availability of openly shared datasets now makes it feasible to perform genuine meta-analyses on empirical data—going beyond traditional literature reviews, which are inherently limited in their ability to account for methodological differences and processing disparities that can influence the aggregation and interpretation of results.

## 5. Functions of consciousness

The second question in Tinbergen’s approach asks what functions consciousness serves. Intuitively, consciousness should have many important functions in our lives. However, several experiments and theoretical arguments challenge this assumption. Theories such as conscious inessentialism and epiphenomenalism argue that consciousness is non-essential for cognitive functions. Conscious inessentialism holds that consciousness is unnecessary for most behaviours, while epiphenomenalism treats it as a byproduct of brain activity without causal influence (see a discussion in [37]). In a similar vein, some evolutionary perspectives suggest that consciousness lacks direct adaptive value, and has been preserved either by chance or as a byproduct of other traits—such as complex brains—that do offer evolutionary advantages [38,39].

For the purposes of our framework, we adopt the hypothesis that consciousness plays a significant functional role in adaptive behaviour. While there is currently no definitive answer to this question, exploring the possible functions of consciousness should guide both empirical and theoretical research. This approach enables testable predictions, supports developmental and cross-species comparisons and links consciousness to underlying neural and cognitive mechanisms. Without this assumption, the study of consciousness risks becoming disconnected from the biological systems it aims to explain. This working hypothesis is important because it keeps consciousness research grounded in empirically testable questions and ensures that the field remains connected to broader issues in behaviour, cognition and evolution.

A neuroethological lens emphasizes why consciousness evolved. A few proposed hypotheses that have been discussed in the literature are as follows:

— *P-consciousness* may have evolved because it serves as:
  (a) a fast, pre-reflective mechanism for monitoring the environment with minimal cognitive overhead—ideal for generating immediate responses in complex sensory landscapes [8].
  (b) a mechanism for attributing intrinsic value to experiences, allowing organisms to compare and prioritize experiences over time [40].
  (c) a mechanism that provides a first-person point of view to guide attention and learning [41,42].
  (d) a mechanism for sexual selection resting upon aesthetic appreciation [43].
— *A-consciousness* may have evolved in species that require the integration of information across sensory modalities for purposes such as communication, reasoning, long-term planning or social coordination—functions commonly observed in social mammals and tool-using birds.

This framework might suggest a continuum of consciousness across species depending on the ecological demands and range of behaviours [44]:

— Species with high *P-consciousness* but limited *A-consciousness* (e.g. some fish, cephalopods or insects).
— Species with both *P-* and *A-consciousness* to varying degrees (e.g. primates, cetaceans, elephants, corvids).

A less explored and recently proposed hypothesis for the function of consciousness is that it may have evolved in part for aesthetic appreciation linked to sexual selection. This was initially implied by Darwin [45] and later reformulated by Richard Prum [43]. Prum introduces the idea of ‘arbitrary coevolution,’ where female preferences and male traits coevolve, leading to elaborate displays and ornaments that may not confer direct survival benefits. This process underscores the significance of subjective experiences and aesthetic appreciation in evolutionary dynamics. For instance, the intricate dances of manakins or the elaborate bowers constructed by bowerbirds are not necessarily indicators of genetic fitness but are products of aesthetic choices. This perspective aligns with the concept of *P-consciousness*, which pertains to the subjective experience of sensory perceptions. In the context of sexual selection, the appreciation of beauty and the resultant mate choices suggest that such subjective experiences can have evolutionary consequences. By valuing traits for their aesthetic appeal, organisms demonstrate that consciousness—particularly the capacity for aesthetic appreciation—can influence evolutionary trajectories. In summary, Prum’s work highlights the integral role of aesthetic appreciation and subjective experience in sexual selection, suggesting that consciousness, through the lens of aesthetic preference, serves a functional role in the evolution of species.

This broader framework challenges the view of consciousness as merely a byproduct of practical, problem-solving functions. Instead, it highlights the complementary roles of *P-consciousness* and *A-consciousness* in evolution. While *A-consciousness* supports goal-directed behaviour, decision making and communication, *P-consciousness* may have evolved to facilitate immediate, affective engagement with the environment—shaping social bonds, aesthetic preferences and reproductive behaviours. Understanding consciousness through this dual lens—both as a tool for adaptive control and as a mechanism for subjective valuation—offers a nuanced and functionally diverse account of its evolutionary origins.

## 6. Consciousness development from fetal to newborn life

Tinbergen’s Question 3 focuses on how a given behaviour—here consciousness—develops in single individuals. Although complex neural networks continue to develop after birth, there is already brain activity before birth that could support basic consciousness [46]. The neural circuitry underpinning the brain’s ability to segregate and integrate information matures as early as the late 2nd trimester of pregnancy [47]. Around week 25 of gestation, thalamocortical connections are formed [48]. These connections are deemed essential for processing sensory input and are considered by many as a foundational step in the development of the neural correlates of consciousness [48]. Functional MRI studies show spontaneous activity in sensory cortices in newborns, indicating early sensory awareness [49–51].

Fetuses and newborns interact with their environment in distinct ways: fetal experiences occur in the womb, which may be crucial for how we start consciously experiencing the self, the body and the world [52]. Contrary to the adult, vision-centric view of consciousness research, early experiences rely heavily on audition and sensorimotor integration. Newborns and toddlers explore the world through touch and movement before their visual systems fully develop [53–55]. Evidence also points to early bodily and emotional awareness: fetuses respond to touch, sound and pain, and newborns show signs of memory, emotional expression and even empathy [48,56–59]. Recognition of the mother’s voice suggests that language-related processing begins prenatally [57,59].

In summary, the current literature supports the idea that consciousness starts developing before birth. Studying consciousness this early in fetuses and newborns offers insights into the early development of sensory awareness, self-perception and fundamental brain mechanisms, which can contribute to a broader understanding of consciousness itself. Moreover, there are important ethical implications, particularly for prenatal care and pain management. For instance, until the late 1980s, surgeries were performed on newborns without anaesthesia owing to misconceptions about their pain perception [60,61]. It is now accepted that the neural pathways for pain perception exist by week 24 of gestation, though debate remains about earlier stages [61].

## 7 Investigating consciousness across species: beyond traditional markers

This section addresses Tinbergen’s Question 4, which focuses on investigating how consciousness has evolved across phylogeny. As scientific understanding advances, the idea that humans are not the only conscious beings is gaining broader acceptance—much like how the field of cosmology eventually recognized that Earth is not the centre of the universe. The challenge now lies in assessing consciousness beyond humans. This is not only ethically important (e.g. in the treatment of animals) but also crucial for understanding how consciousness may have evolved independently of high-level cognitive functions. Traditionally, consciousness in non-human animals has been inferred through specific cognitive and behavioural markers—such as working memory, endogenous attention, selective learning and goal-directed behaviour, among others [15,62–66]—which are often tested using highly controlled tasks like the Delayed Matching to Sample (DMS [67–76]) or Posner’s cueing paradigm [77–87]. These tasks have provided strong evidence for conscious processing in mammals and birds [67–104], but show mixed or limited success in other species such as reptiles, amphibians, fish and insects [62,105–110].

However, such paradigms often lack functional ethological validity—they require extensive training and may not align with the animal’s natural behaviours or motivations. Poor performance may instead reflect task irrelevance or stress and not absence of consciousness. Further complicating the issue, the link between these markers and consciousness is not definitive; non-conscious working memory, for example, has been documented in humans [111].

This challenges the assumption that a single cognitive profile or neural architecture may underlie consciousness universally. In fact, some evidence suggests that a diversity of neural substrates may support conscious experience across species. For example, while mammals possess a neocortex, birds achieve similar cognitive functions using a different brain organization, suggesting that a neocortex is not necessary for consciousness [112]. Research by Feinberg & Mallatt supports this view, proposing that a minimal neural architecture for sensory consciousness and *qualia* in vertebrates may include a forebrain (but not necessarily a developed cerebral cortex), midbrain and hindbrain [113]. This implies that consciousness could arise from different neural substrates and may have evolved independently in various animal groups [114]. Feinberg & Mallatt suggest a two-step evolutionary history: first, the optic tectum served as the centre of multi-sensory consciousness (in fish and amphibians), followed by a shift to the dorsal pallium or cerebral cortex (in mammals, reptiles and birds) [113]. According to their view, the lamprey—a group of jawless fish—meets these criteria, making it the simplest extant vertebrate with sensory consciousness and qualia [113].

A strategy to move beyond anthropocentric constraints is to embrace a multidimensional and ecologically grounded approach. Instead of posing the binary question ‘Is this species conscious?’, we could ask: ‘How does consciousness manifest in this species, in what form, and serving what function?’ Naturalistic behaviours—such as social interaction, play, pain responses, motivational trade-offs, self/others distinction and even mirror self-recognition—may offer more ecologically valid indicators of conscious experience [115–126]. For instance, bees tolerate aversive conditions to obtain higher rewards [119], octopuses exhibit centralized processing of nociceptive signals consistent with pain [118] and cleaner fish have passed versions of the mirror test [126]. The latter is particularly striking, as self-recognition had been presumed exclusive to ‘higher’ mammals. These behaviours suggest that *P-consciousness*—the felt, affective aspect of experience—is widespread and functionally significant. Meanwhile, *A-consciousness*—which involves reportable, integrated, goal-directed cognition—may be more variable and evolved to meet specific ecological or social demands in certain taxa.

Recognizing the complexity and potential diversity of conscious experiences across species calls for a framework that captures variation without relying on a single, human-centric benchmark. One such model is Birch’s multidimensional framework [127], which outlines five key dimensions of consciousness: perceptual richness (detail of environmental perception), evaluative richness (emotional complexity), integration at a time (unified experience), integration across time (temporal continuity) and self-consciousness (awareness of self). Each dimension can be operationalized through specific behavioural and physiological subcomponents and applied across species with diverse sensory modalities and cognitive architectures. This allows for a more inclusive and flexible understanding of consciousness—where each species may have a unique profile, rather than being ranked along a single continuum.

While this framework focuses on proximate mechanisms—those observable in individuals—it also opens new possibilities for comparative research. Mappingrelated species across these dimensions could yield insights into the evolution of consciousness and its adaptive value. In doing so, it shifts the question from *whether* an animal is conscious to *how* and *why* consciousness appears in diverse forms.

## 8. A neuroethological approach to consciousness

To move beyond the current, narrow focus on adult humans in consciousness research, we have laid out a broader investigative strategy based on Tinbergen’s four-question framework for studying animal behaviour. We term this a ‘neuroethological approach’ to consciousness, as it integrates insights from neurophysiology with the comparative study of naturalistic, species-specific behaviours across diverse taxa. While we are not the first to advocate for a neuroethological perspective on consciousness [128,129], prior versions emphasized different aspects of consciousness. What distinguishes our approach is the explicit incorporation of Tinbergen’s framework, which systematically addresses questions of mechanism, ontogeny, function and phylogeny. A key strength of this framework is that each question can serve as a constraint on the others, enabling an iterative process of theoretical refinement. For example, the question of mechanism can help identify the earliest developmental point at which a candidate neural or cognitive feature appears, providing insights into ontogeny. This is especially valuable in humans, where we know consciousness eventually emerges. Since human infants share much of their neural circuitry and ecological context with adults, we can approach the problem in two stages. First, from adults to infants, tracing the developmental trajectory of consciousness allows us to pinpoint when latent potential gives rise to actual expression. This can reveal which features are added—or perhaps even lost—over time. Some dimensions of consciousness may be present early (and conserved across species), while others emerge later; for example, multisensory integration may follow the development of unimodal sensory processing, with basic tactile awareness (e.g. as revealed by somatosensory protomaps) possibly preceding the maturation of auditory and visual systems. Second, from humans to other species, developmental trajectories in humans offer testable hypotheses that can guide the search for analogous features in non-human animals. This comparative, cross-species approach fosters an iterative process in which findings in one species inform investigations in another—narrowing the search space for identifying consciousness-related mechanisms and functions across the animal kingdom.

**Figure 1.**
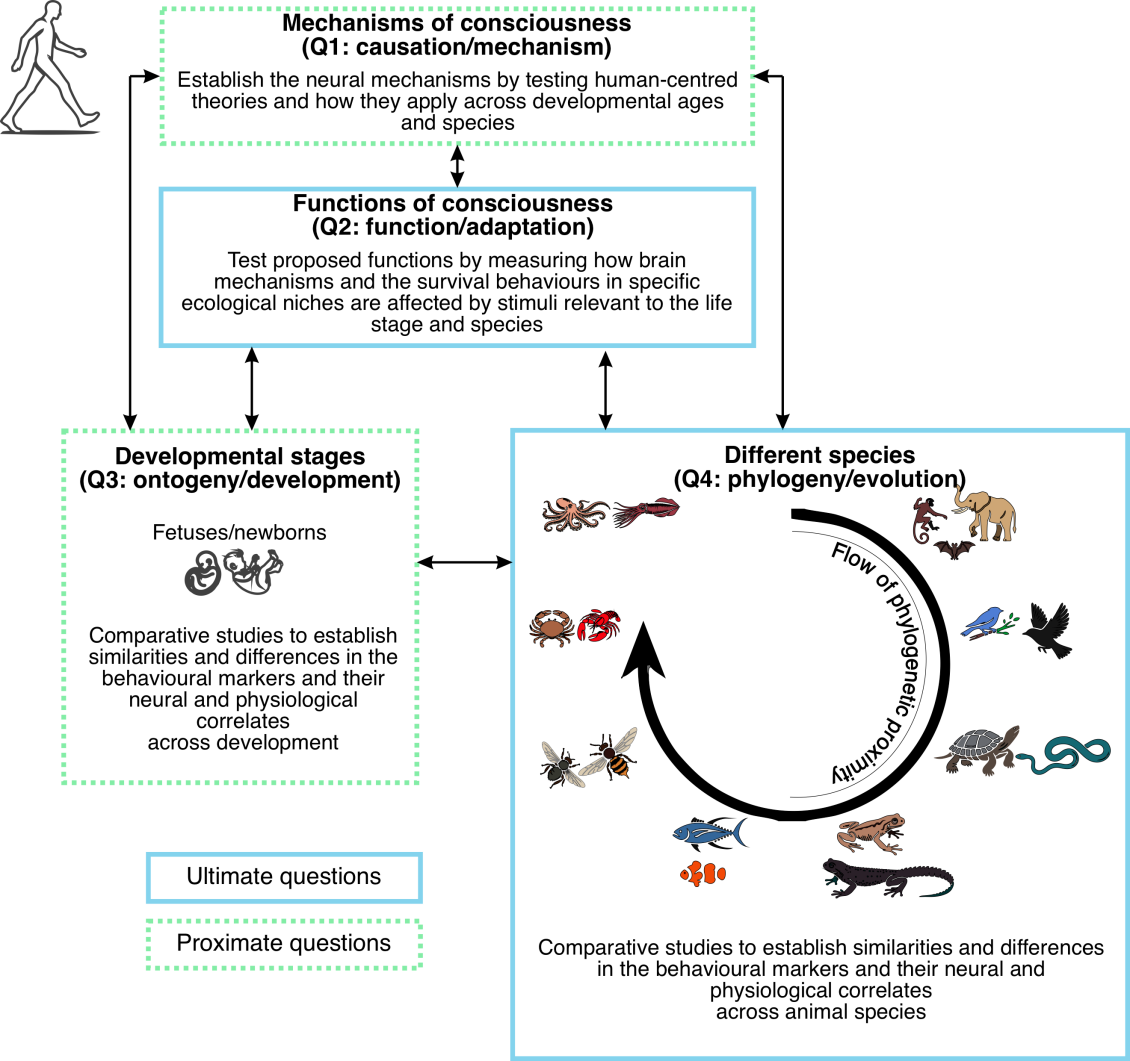
Neuroethological framework to study consciousness adapting Tinbergen’s four questions [6]. Double-headed arrows illustrate that each question can serve as a constraint on the others, enabling an iterative process of theoretical refinement.

Having discussed the Tinbergen questions throughout this work, in this final section, we illustrate how they can be applied to identify and evaluate consciousness in animals and fetuses (figure 1). We begin with Question 1: Mechanism (figure 1, top). To explore the underlying neural mechanisms of consciousness, we can test whether human-centred theories, such as the Attentional Schema Theory (AST [130]). AST emphasizes the role of selective attention in conscious experience. Using modern neurophysiological tools, we can identify brain regions and circuits involved in attention, from high-level areas like the frontal and parietal cortices to subcortical regions such as the superior colliculus, thalamus and reticular formation [131–133]. To extend this analysis across taxa, one would record from analogous structures in distantly related clades such as arthropods and cephalopods, presenting them with ecologically relevant sensory stimuli to engage attention. Crucially, these recordings must occur during behaviours natural to the animal, thereby preserving ecological validity—the hallmark of a truly neuroethological approach.

Next, using the information on mechanisms just attained, we proceed to Question 2, which seeks the adaptive functions of consciousness. Continuing to use our example of selective attention, we would investigate how such attention is modulated by survival tasks requiring dynamic prioritization of sensory input (e.g. foraging or predator detection). Again, the brain’s activities will be recorded with modern neurophysiological techniques, but now the mechanisms will be evaluated by how they differ across ecological contexts. During the recordings, the associated survival *behaviours* will be monitored to shed light on the adaptive functions of the experiences. In addition, the proposed brain areas for each function can be electrically stimulated to see if this invokes a behavioural response reflects that function.

Turning to Question 3, developmental studies could assess how the attentional mechanisms resulting from researching Question 1 (see above) emerge in infants and mature over time, providing insight into the developmental trajectory of consciousness. For Question 4, by conducting comparative studies, we could examine how specific neural markers of selective attention, such as oscillatory activity or recurrent processing, vary across species according to the species’ phylogenetic proximity—whether they are closely or distantly related. This would allow us to identify both conserved and species-specific mechanisms, shedding light on the possible evolutionary history and advantages of consciousness.

Together, this comparative, multi-species framework could provide a powerful foundation for developing a robust, biologically grounded theory of consciousness. While direct access to another organism’s subjective experience remains challenging, the question is not whether we can fully understand what it feels like to be another creature, but rather: what does consciousness—across its many gradations and forms—do for living agents within the ecological niches in which they have evolved? This shift in perspective, from asking who is conscious to asking how and why consciousness manifests, may moves us closer to unravelling one of the deepest scientific mysteries of the twenty-first century.

## Data Availability

This article has no additional data.
